# Comparing the Through-Thickness Gradient of the Deformed and Recrystallized Microstructure in Tantalum with Unidirectional and Clock Rolling

**DOI:** 10.3390/ma12010169

**Published:** 2019-01-07

**Authors:** Jialin Zhu, Shifeng Liu, Xiaoli Yuan, Qing Liu

**Affiliations:** 1 College of Materials Science and Engineering, Chongqing University, No. 174 Shazheng Street, Shapingba District, Chongqing 400044, China; jialinzhu@cqu.edu.cn; 2 College of Metallurgy and Material Engineering, Chongqing University of Science and Technology, Chongqing 401331, China; yuanxiaoli1981@126.com

**Keywords:** tantalum, clock rolling, microstructure gradient, schmid factor

## Abstract

Controlling the microstructure homogeneity is crucial in achieving high quality tantalum (Ta) sputtering targets used in integrated circuit fabrication. Unluckily, traditional rolling easily generates a microstructure gradient along the thickness direction in Ta sheets. The deformation and recrystallization behavior of unidirectional and clock rolled Ta with an 87% strain were therefore systematically compared to investigate whether the change of strain-pass can effectively ameliorate the microstructure gradient along the thickness. Electron backscatter diffraction was used to analyze the misorientation characteristics of the deformed grains. A strong microstructure gradient exists in the unidirectional rolled (UR) sheets. Many microshear bands and well-defined microbands occurred in {111} deformed grains in the UR sheets, especially in the center region, while the grain fragmentation with {111} and {100} orientation in the clock rolled (CR) sheets was more homogenous along the thickness. The kernel average misorientation (KAM) and grain reference orientation deviation-hyper (GROD-Hyper) further confirmed these differences. X-ray line profile analysis (XLPA) indicated that the stored energy distribution was more inhomogeneous in the UR sheets. Schmid factor analysis suggested that the strain path changes due to clock rolling promoted the activation of multiple slip systems in {111} oriented grains. Upon static annealing, homogeneous nucleation combined with a slower grain growth rate resulted in finer and more uniform grain size for the CR sheet. In contrast, a strong recrystallization microstructure-gradient along the thickness formed in the UR sheets, which is attributed to the fact that the higher stored energy and more preferential nucleation sites led to faster recrystallization in the center region, as compared with the surface region. Thus, clock rolling can effectively improve the homogeneity of the through-thickness recrystallization microstructure of Ta sheets.

## 1. Introduction

Tantalum, a transition metal with a body-centered-cubic (BCC) structure, is widely used in the integrated circuit industry (IC), for example, as sputtering targets [1]. The properties of deposited thin films are determined by the performance of the sputtering targets during the sputtering. Sputtering effectiveness depends highly on the crystallographic orientation (generally referred to as texture) of each grain due to different densities of atomic plane, and texture clusters have unfavorable effects on the uniformity of films [2,3]. Besides, the grain size also plays an important role in the sputtering rate. Targets with fine grain size enable a higher deposition rate than those with large grain size because grain boundaries are more readily attacked during sputtering [4]. Therefore, homogenous microstructure and random texture distribution along the thickness are critical for assuring reliable sputtering performance of Ta.

Unluckily, the texture and microstructure may be inhomogeneous through the thickness of rolled plates due to the inhomogeneity of deformation during rolling, especially for materials with high stacking fault energy, such as aluminum or its alloys with face-centered-cubic (FCC) structure, interstitial free (IF) steel, or high purity Ta with body-centered-cubic (BCC) structure [5,6,7,8,9]. It is accepted that the deformation zone parameter *l*/*h* (where l is the length of contact between the rolls and the specimen, and h is the average thickness of the sample for each rolling pass) and the friction between the roll and the sheet surface influence the texture evolution in different thickness layers due to the change in the stress–strain state [10,11,12]. Clark and co-works reported that rolling with a deformation zone parameter of 0.67~1.25 showed severe through-thickness texture gradients, but less severe texture gradients were obtained when the deformation zone parameter was within the range from 1.06 to 2.70 [13]. Mishin et al. [14] found that grains in different layers have morphologically different microstructures in aluminum when rolled with a small deformation zone parameter. In addition, the initial through-thickness microstructure gradient prior to rolling is another factor affecting the homogeneity of rolling texture and through-thickness texture variation in cold-rolled Ta sheets. For example, processing of Ta ingots produced by electron beam melting (EBM) generated severe texture gradients along the thickness [13,15].

The grain size, texture evolution, dislocation arrangement, and recrystallization kinetics of metal materials are highly dependent on their primary deformation process, such as clock rolling, asymmetrical rolling, and cross-roll rolling. For example, the cross rolling introduced by Oertel et al. [16] was conducted on molybdenum plates to enhance the metal’s formability due to the developed homogeneity of texture and microstructure refinement. Asymmetrical rolling was adopted by Hamad et al. [17] to obtain equiaxed ultrafine ferrite grains in 0.18 wt % carbon steel. The cross-roll rolling was applied by Kim et al. [18] to manufacture AZ31 Mg sheets with uniform texture intensity from the surface layer to the middle layer. Recently, work by our team reported that 135° clock rolling—sequentially changing the rolling direction by 135° around the normal direction—was effective in alleviating the through-thickness texture gradients and orientation dependence that often exist in cold-rolled Ta plates [15,19,20]. However, the distribution of deformed and subsequent recrystallized microstructure along the thickness has not been scrutinized in more detail.

Undoubtedly, the strain distribution induced by clock rolling will influence the microstructure (e.g., grain size, dislocation density, orientation, etc.) within different deformation layers. Such microstructure gradients greatly affect the preferential nucleation location and then influence the homogeneity of the recrystallization microstructure of the annealed clock rolled (CR) sheets. The current work investigated whether 135° clock rolling can effectively improve the deformed and recrystallized microstructure gradient along the thickness when compared with unidirectional rolling, and if yes, how.

## 2. Materials and Methods

A high-purity (99.95 wt %) tantalum ingot was obtained by electron beam melting and the chemical composition of the ingot is shown in Table 1. The initial tantalum ingot was processed by forging to a 20-mm thickness, followed by annealing. Two groups of samples were prepared from the same ingot and were rolled to 2.8 mm (87% reduction) in thickness in 16 rolling passes in total by two rolling schemes: unidirectional rolling and 135° clock rolling, The detailed working process and rolling parameters are presented elsewhere [20]. Note that all the rolling experiments were carried out at room temperature without lubricant, and the rolling speed was 0.2 m/s. The value of deformation zone parameter was set between 2 and 3.7 for each rolling pass, which is believed to guarantee a plane-strain state [15]. The two groups of rolled specimens were annealed under vacuum at 1065 °C for different times to capture the early stage of recrystallization and then were immediately quenched into cold water.

The microstructure of the deformed and annealed specimens was characterized by an electron backscatter diffraction (EBSD) system attached to a TESCAN MIRA 3 field emission gun-scanning electron microscope. The longitudinal section of the specimens (i.e., the plane containing the normal direction (ND) and the rolling direction (RD)) was mechanically and electrolytically polished for the EBSD characterization. These scans were conducted on both the surface and center regions, and it is worth mentioning that at least five maps were taken for each condition to ensure the reliability of the data. The dislocation morphology was observed using a JEOL JEM-2100 transmission electron microscope (TEM) with an operating voltage of 200 kV. Foil preparation was in accordance with the method suggested by Liu et al. [21].

Macrotextures and bulk stored energy of the deformed sample were measured by X-ray diffraction (XRD, Rigaku D/max-2500 PC) with a Cu Kα radiation. Four incomplete pole figures, {110}, {200}, {211}, and {222}, were measured by the Schultz back reflection technique, and the pole distance angle ran from 20° to 90° with a step size of 5°. The orientation distribution functions (ODFs) were calculated using the Arbitrarily Defined Cells (ADC) method [22].

XRD measurements can also be used to evaluate the lattice distortion, i.e., the stored energy. The presence of internal residual stress, causing large variation in the lattice spacings, can lead to the broadening of X-ray diffraction lines [23]. Rajmohan et al. [24] have adopted a modified Stibitz formula [25] for calculating the stored energy along different crystallographic orientations using a direction-dependent Young’s modulus *Y*_hkl_ and Poisson’s ratio *v*_hkl_ as follows:

(1) $$SE_{hkl}=\frac{3}{2}Y_{hkl}\frac{{\left(△d/d\right)}^{2}}{\left(1+2\nu_{hkl}^{2}\right)}$$

where *Y*_{hkl}_ and *v*_{hkl}_ are the direction-dependent Young’s modulus and Poisson’s ratio, which are 145.517 GPa and 0.316 for the θ fiber and 387.931 GPa and 0.362 for the γ fiber, respectively [26,27]. The relative change in the lattice spacing (∆*d*/*d*) can be acquired form the broadening of diffraction peaks from the following relation:

(2) $$△d/d=\frac{\sqrt{B_{r}^{2}-B_{a}^{2}}}{2\tan\theta }$$

where *θ* is the Bragg angle and *B_r_* and *B_a_* are the measured full width at half maximum of the rolled and fully recrystallized Ta at the diffraction angle 2*θ*, respectively. To quantify the orientation-dependent bulk stored energy, the {200} and {222} line profiles were recorded by step-scan with a step size of 0.01° and timing of 1 s per step. An acceptable peak was obtained after optimizing the peak-to-background ratio.

To evaluate the recrystallization process, Vickers hardness (VH) measurements were performed on an MH-5L model hardness tester, and a load of 300 g with a dwell time of 10 s were employed. In order to ensure the accuracy of statistical analysis, at least 15 indentations in the line path were made for each sample to obtain an average value. The examinations of the positions were conducted on the rolling plane of the specimens (i.e., the plane containing the RD and transverse direction (TD)) in the surface and center regions.

## 3. Results

### 3.1. Rolling Texture

**Figure 1:**
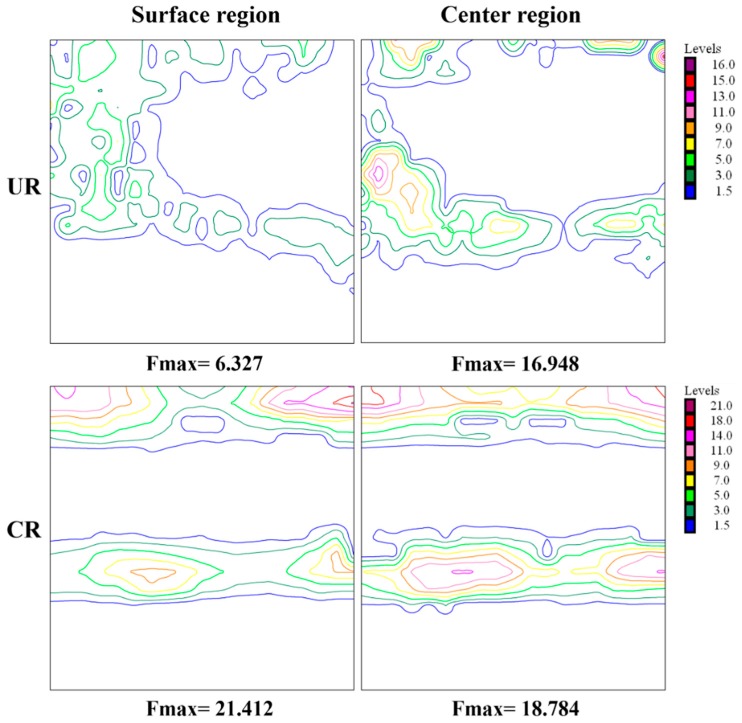
Orientation distribution function (ODF) sections (φ_2_ = 45°) at the surface and center region for unidirectional rolled-Ta (UR-Ta) and clock rolled-Ta (CR-Ta).

Generally, the rolling texture of BCC metals is mainly concentrated into three components: α fiber with <110> parallel to the RD, γ fiber with <111> along the normal direction (ND), and θ fiber with <100> along the ND [28]. All the orientations along these fibers can be revealed in the φ_2_ = 45° section in the Euler space, as illustrated in Reference [20]. Figure 1 shows the texture distribution in the surface and center regions measured by XRD for unidirectional rolled (UR) and CR Ta. As shown in the ODF sections, texture types through the thickness for UR-Ta are almost the same, consisting of the θ, α, and γ fibers, but the ODF intensity in the center region is much stronger. However, there is a weak difference in ODF intensity and orientation variation along the thickness for CR sheets, which is consistent with the result by Fan et al. [15]. In addition, more complete {111} and {100} orientation along the γ and θ fibers were obtained by clock rolling. It should be noted that the rotation of the specimen about the ND in clock rolling leads to the continuous change of grain orientation relative to RD, which causes the α-fiber to almost disappear [29]. Besides, more slip systems could be activated due to the change of strain-path during clock rolling, and random grains then undergo a reorientation and gradually flow into the two ideal deformation fibers [28].

### 3.2. Bulk Stored Energy

**Figure 2:**
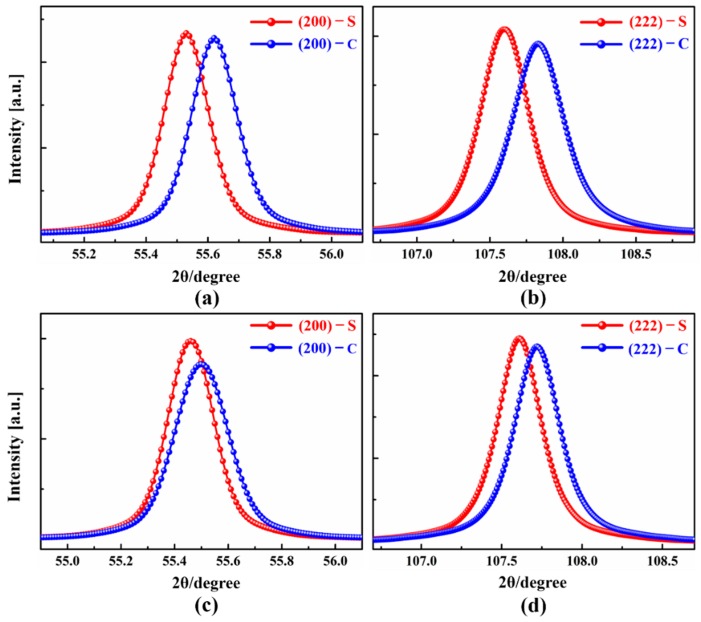
Fitted {200} and {222} diffraction peaks at the surface (S) and center region (C) for UR-Ta and CR-Ta, respectively. (**a**) UR, {200}; (**b**) UR, {222}; (**c**) CR, {200}; and (**d**) CR, {222}.

Figure 2 shows the fitted diffraction peaks and the values of the stored energy along the {200} and {222} planes estimated using the direction-dependent *Y*_{hkl}_ and *v*_{hkl}_ as presented in Table 2 for the UR and CR sample. It can be seen that the bulk stored energy was highly orientation-dependent for UR-Ta, and the value of the stored energy was higher in the {111} matrix, as compared to the {100} matrix. It has also been demonstrated that the energy stored in the {111} matrix in low carbon steel was significantly higher than that of the {100} matrix after a rolling reduction of 70%. While the energy stored in the {111} matrix was significantly lowered, it increased moderately in the {100} matrix for CR-Ta. In other words, the clock rolling effectively narrowed the bulk stored energy gap between the {111} and {100} matrices. Moreover, the stored energy was distributed more homogenously across the thickness.

### 3.3. Deformed Microstructure 

**Figure 3:**
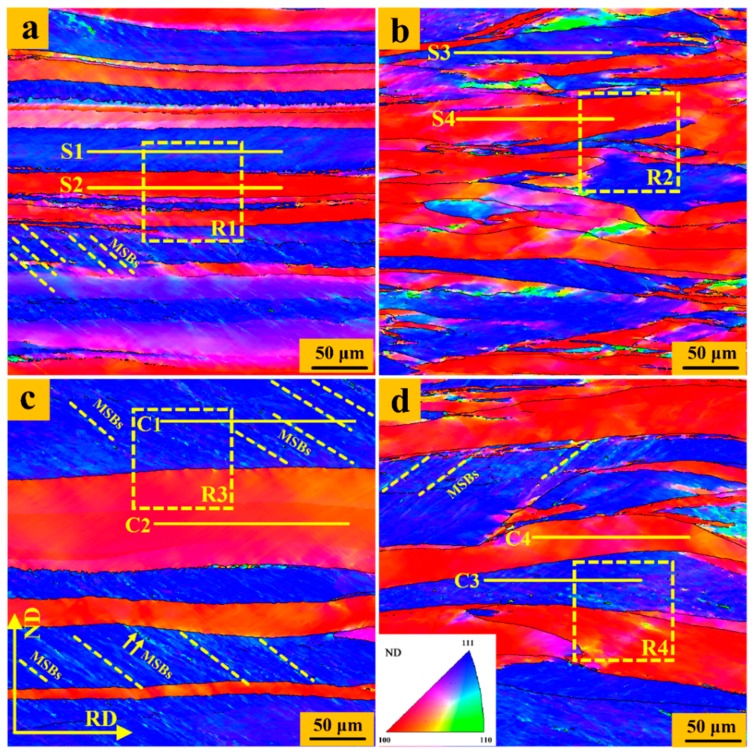
Inverse pole figure (IPF) maps. UR: (**a**) surface region and (**c**) center region, CR: (**b**) surface region and (**d**) center region. Step size 0.25 μm.

The microstructure of the UR and CR samples through the thickness is presented using an inverse pole figure (IPF) map [30,31]. The grains in the IPF map are colored according to the orientation of the sample’s ND in a standard triangle of crystal-stereographic projection, as indicated in Figure 3d. Obviously, the micro-texture from the EBSD results indicate that the {100}<uvw> (<100>//ND) and {111}<uvw> (<111>//ND) are typical textures, which are in a good agreement with the macro-texture measured by XRD (Figure 1). The {111} and {100} elongated matrices arranged more locally and more densely in the surface region of sample. It is worth noting that there is a significant difference in morphology of grain boundary between the UR and CR samples. The rolled grains of the UR sample have relatively straight and planar grain boundaries. The grain boundaries in the CR sample show a certain degree of bending and fluctuation along the RD direction, and even that some {111}–{100} grain boundaries infiltrate and intertwine with each other.

**Figure 4:**
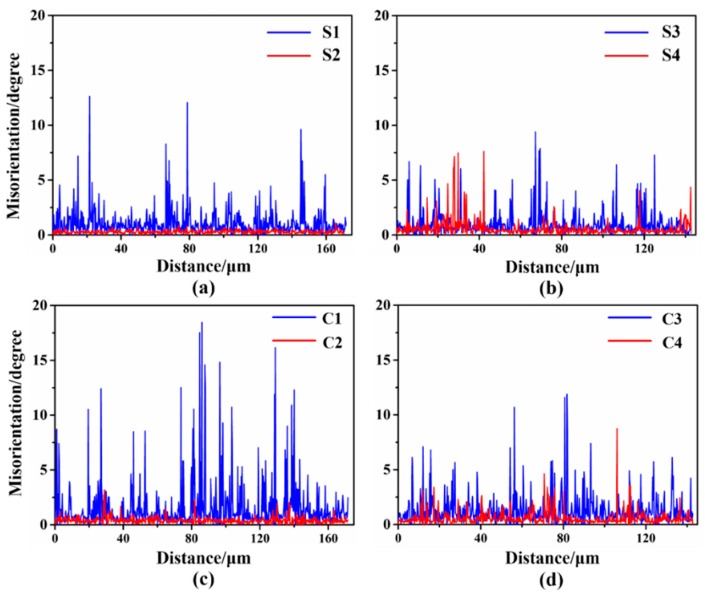
Misorientation maps along the scanning lines ‘S1’ to ‘S4’ and ‘C1’, ‘C2’, to ‘C4’, as shown in Figure 3. UR: (**a**) surface region and (**c**) center region, CR: (**b**) surface region and (**d**) center region.

Figure 4 shows the ‘point to point’ (PTP) misorientation angle distribution along the scanning lines ‘S1’ to ‘S4’ and ‘C1’ to ‘C4’, respectively, which reveals the orientation difference between neighboring points. Many micro shear bands (MSBs), which usually appear with the {110} <uvw> (<110>//ND) orientation [32], delineated by yellow oblique lines exist in the {111} matrix in the center region of the UR sheets. In addition, peaks in the boundaries of the MSBs in the {111} matrix in the UR-Ta show a higher angle, exceeding 15°, in the PTP plot. The PTP misorientation-angle in the {100} matrix (profile ‘S2’ and ‘C2’) is much lower. As for the CR-Ta, peaks of the PTP profile in the {111} and {100} matrix are in general lower than 10° and few sharp peaks occur in the CR sheets, even in the {111} matrix. More accurately, deformation in the interior of the {111} and {100} matrices is relatively homogeneous along the thickness.

**Figure 5:**
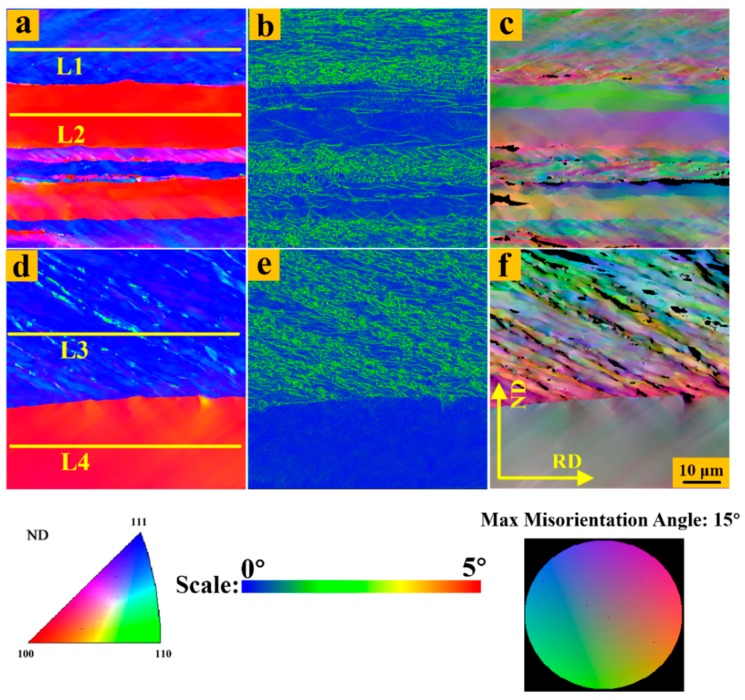
Microstructure of 87% UR-Ta in the regions ‘R1’ and ‘R3’, respectively. ‘R1’: (**a**) IPF map; (**b**) kernel average misorientation (KAM); (**c**) grain reference orientation deviation-hyper (GROD-Hyper). ‘R2’: (**d**) IPF map; (**e**) KAM; (**f**) GROD-Hyper.

**Figure 6:**
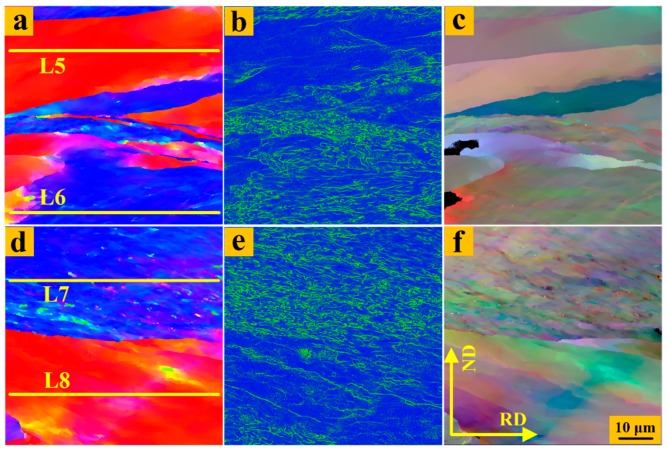
Microstructures of 87% CR-Ta specimens in regions ‘R2’ and ‘R4’, respectively. ‘R2’: (**a**) IPF map; (**b**) KAM; (**c**) GROD-Hyper. ‘R4’: (**d**) IPF map; (**e**) KAM; (**f**) GROD-Hyper. Step size: 60 nm.

To better compare the local microstructure and expose the degree of grain splitting of UR and CR sheets through the thickness, the kernel average misorientation (KAM) and grain reference orientation deviation-hyper (GROD-Hyper) were adopted, as shown in Figure 5 and Figure 6. It should be noted that KAM is the average misorientation between a point on the measurement grid and its nearest neighbor points, and the closer to blue that the color is, the lower the local-misorientation becomes. GROD-Hyper represents the deviation between a measuring point and the average orientation of a grain, and the closer the color is to the area on the outside of the circle, the higher the deviation [33]. The local stress distribution in the interior of the grain can be well matched with the local-misorientation. The matching depends on the fact that each point in the EBSD scan has its own individual value of local-misorientation, and this technique uses fine scanning and high-resolution microscopes. High KAM values in the {111} matrix and low KAM values in the {100} matrix in the UR sheets were observed, especially in the center region. In other words, the deformation was extremely inhomogeneous for the UR sheets through the thickness, the fragmentation of the {111} matrix in the center region was more serious, and the internal stress stored in the {111} matrix was higher. For GROD-Hyper, the color was bright in the {111} matrix while the color was dim in the {100} matrix in the UR-Ta, which means that the dispersion was larger and more substructures appeared in the {111} matrix, which is in accordance with the occurrence of internal stress. The colors of the {111} and {100} matrices in the surface and center regions in the CR-Ta were close to those on the inside of the circle, indicating a more uniform deformation along the thickness. In addition, the higher the internal stress within grains, the greater the stored energy. Therefore, clock rolling was effective in homogenizing the stored energy distribution in Ta sheets along the thickness.

### 3.4. Microstructure Evolution during Annealing

**Figure 7:**
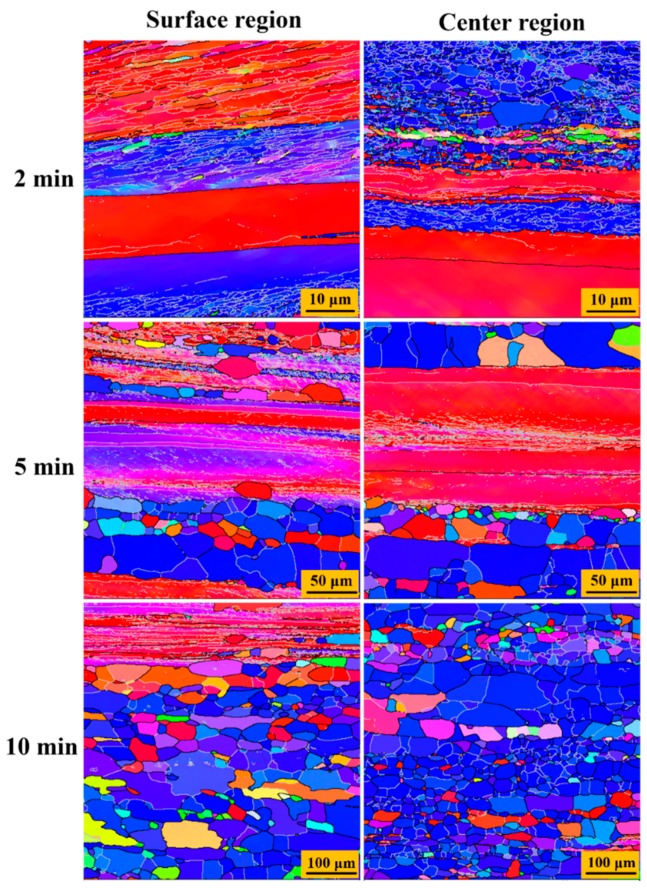
The evolution of the microstructure of the 87% UR sample annealed for different times at 1065 °C. Step size 0.1 μm for 2 min, step size 0.6 μm for 5 min, and step size 1.5 μm for 10 min.

**Figure 8:**
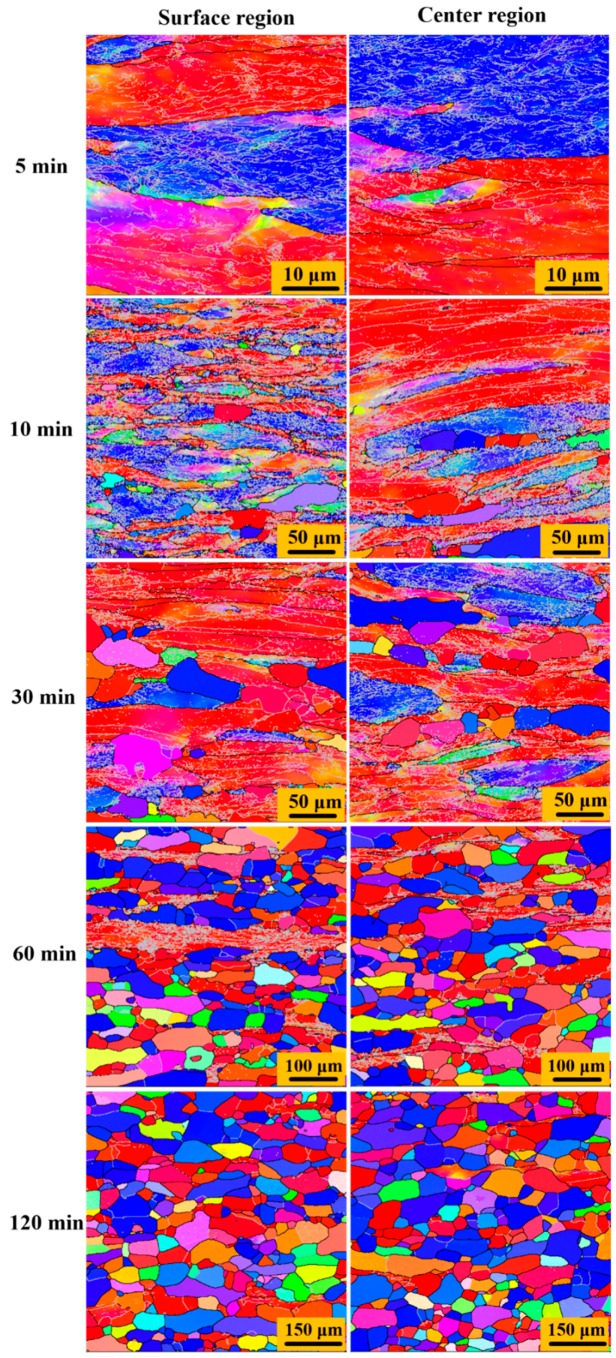
The evolution of the microstructure of the 87% CR sample annealed for different times at 1065 °C. Step size 0.1 μm for 5 min, step size 0.6 μm for 10 min and 30 min, step size 1.5 μm for 60 min and 120 min.

**Figure 9:**
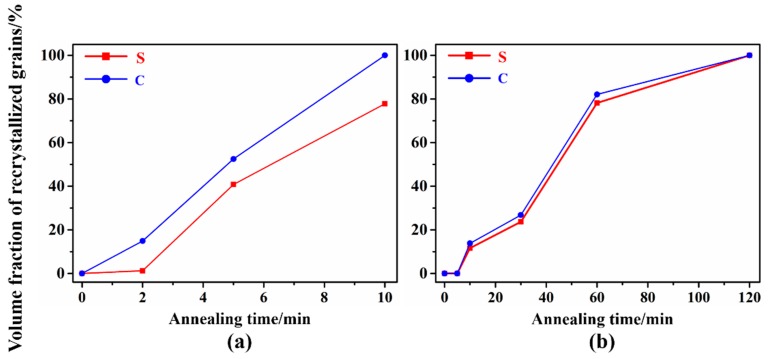
The volume fractions of recrystallized grains with annealing times at the surface and center regions of the UR and CR-Ta samples: (**a**) UR-Ta; (**b**) CR-Ta.

Figure 7 and Figure 8 show the microstructure evolution of 87 % UR-Ta and CR-Ta along the thickness during annealing at 1065 °C for different times. Figure 9 shows the volume fractions of recrystallized grains with the annealing time in the surface and center regions of UR and CR-Ta samples. It can be noted that the grain boundaries with misorientation angles higher than 15° are represented with dark solid lines. While the sub-grain boundaries, or sub-boundaries, with misorientation angles higher than 2° and less than 15° are depicted by silver solid lines. As explicitly presented in Figure 7, some new dislocation-free grains with {111} orientation appeared within the {111} deformed grains in the center region of the UR-Ta when annealled for 2 min, while grains in the surface region were in the deformed state. After annealing for 5 min, the {111} deformed matrix in the center region recrystallized completely at first, whereas a few elongated {111} grains still existed in the surface region, meaning a partial recrystallized state. By further extending the annealing time to 10 min, the {100} deformed matrix was also consumed fully and the center region was in a complete recrystallization state. A few elongated {100} grains were still composed of some sub-boundaries and remained deformed in the surface region. It is obvious that center region recrystallized significantly faster than the surface for UR-Ta. As for the CR-Ta, no recrystallized grains emerged from the deformation matrix through the thickness after annealing for 5 min. Many new defect-free grains appeared at the {111}–{100} boundaries along the thickness when annealing time reached 10 min. When annealing time increases to 60 min, only some {100} deformed matrices can be distinguished from the surface and center regions. By extending the annealing time to 120 min, the annealed microstructure through the thickness consisted of equiaxed grains with relatively random orientation as compared to the UR-Ta, and it was almost free of sub-boundaries, indicating a fully recrystallized state. Apparently, clock rolling is conducive to homogeneous recrystallization along the thickness, while recrystallization kinetics for the UR sheets at different layers are not consistent, and the UR sheets show much easier and faster recrystallization than the CR sheets, as shown in Figure 9.

### 3.5. Microhardness

**Figure 10:**
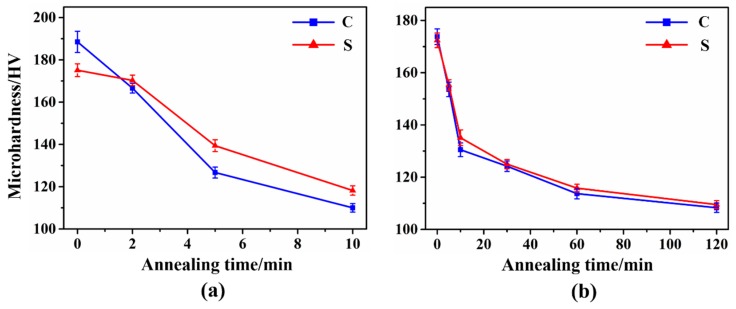
Evolution of micro-hardness of UR and CR-Ta samples as a function of annealing time in the surface and center regions: (**a**) UR-Ta; (**b**) CR-Ta.

Microhardness measurement is an effective method to investigate the changes in mechanical properties and, in particular, it can easily be related with the degree of recrystallization of a metal [34,35]. Figure 10 depicts the microhardness evolution of UR and CR-Ta annealed at 1065 °C for different holding times. The center region of the deformed UR-Ta showed higher microhardness than the surface. After annealing, the microhardness of the center region dropped much more quickly than that of the surface. It is worth noting the hardness value in the surface region was a little higher than that at the center after annealing for 10 min, and the difference between them reached 8.2 HV because of the existence of the {100} deformed matrix. As for the CR-Ta, there was a dramatic drop in the hardness curve after annealing for 10 min, suggesting the occurrence of recovery or the onset of recrystallization. When samples were annealed for 60 min, a further decrease in the curve was observed. After that, the variation in hardness was in a slow trend and reached a minimum after 120 min, evidencing that the deformed microstructure was substituted by new defect-free recrystallized microstructure. The hardness curves of the surface and center regions are nearly consistent and show relatively homogeneous recrystallization kinetics when compared with UR-Ta.

## 4. Discussion

### 4.1. Orientation-Dependent Grain Subdivision

Since the grain subdivision or fragmentation intrinsically depends on activated slip systems, it is reasonable to assume that clock rolling has introduced some latent slip systems. To further analyze and clarify the discrepancy in deformation behavior between UR and CR sheets along the thickness direction, the Schmid factors were applied to judge the activation of different slip systems. As is clearly presented in Figure 5 and Figure 6, 78 points were selected at random (624 points in total) along each line, L1–L8, which are marked in the {111} and {100} matrices. Then, the Schimd factors of each slip system for these points were computed based on the following equation [36] and some are presented in Table 3.

(3) $$SF_{rolling}=0.5\times \left(\cos\alpha \times \cos\beta -\cos\gamma \times \cos\delta \right)$$

where *α* (or *β*) is the angle between the rolling direction and the slip plane normal (or slip direction); while *γ* (or *δ*) is the angle between the normal direction and the slip plane normal (or slip direction). It is noted that for the parameter (*S_M_* − *S_S_*) × *S_M_*^−1^, the Schmid factor difference ratio (SFDR) between *S_M_* and *S_S_* is used to illuminate how a slip is selected as the primary slip system.

**Figure 11:**
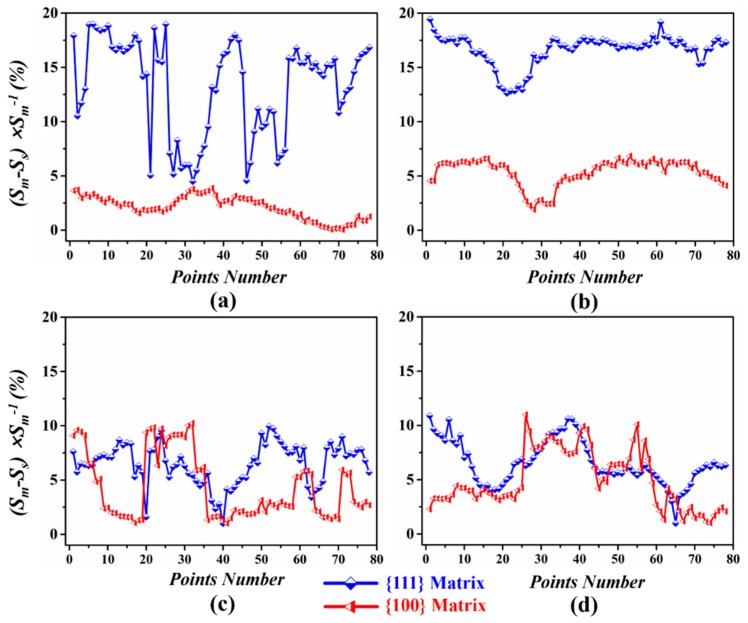
Schmid factor difference ratio (SFDR) values for 624 points selected in lines varying from ‘L1’ to ‘L8’. (**a**) For the points at the surface of the UR-Ta; (**b**) For the points at the center of the UR-Ta; (**c**) For the points at the surface of the CR-Ta; (**d**) For the points at the center of the CR-Ta.

**Figure 12:**
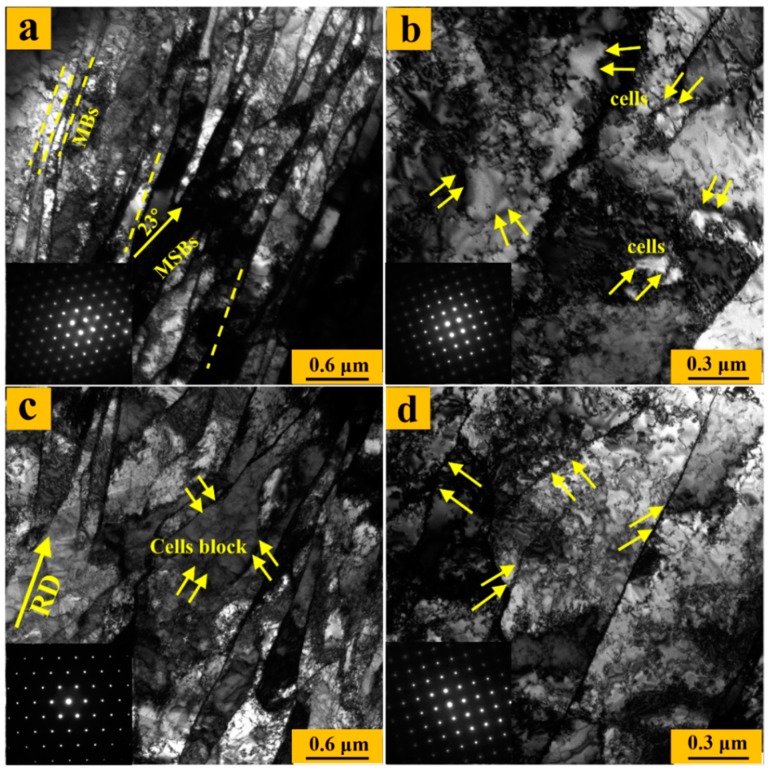
TEM images of the UR and CR samples. UR: (**a**) {111} matrix and (**b**) {100} matrix. CR: (**c**) {111} matrix and (**d**) {100} matrix.

The Schmid factor was calculated for the {110} <111> and {112} <111> slip systems of the body-centered cubic lattice with the help of matlab. The SFDR value for a point was applied to explore the probability of activating one or more (two) slip systems in this point. More accurately, large SFDR values represent only one-slip system that can be easily activated, while more slip systems are prone to actuate if the SFDR value is small. Obviously, most of points in the {111} matrix in the UR sheets, especially in the center region, had comparatively large SFDR values, as showed in Figure 11, while the {100} matrix possed relatively small SFDR values. A slip more active than others would lead to intensive shear localized on a one-slip plane upon rolling and make a contribution to the formation of a severely deformed substructure in the {111} matrix of the UR sheets [37], which fits well with the fact that many straight MBs (a parallel high dense dislocation walls) and MSBs occurred in the {111} matrix in the UR sheets, as shown in Figure 12a. In contrast, only some dislocation cells appeared in the {100} matrix, as can be distinctly observed in Figure 12b. The huge difference in dislocation morphology and density caused by the difference in SFDR values, particularly in the center region, surely enlarged the stored-energy gap between the {111} and {100} matrices.

As for CR-Ta, deformation was relatively homogeneous and multiple slip systems appear to activate in both the {111} and {100} matrices due to the relatively low SFDR value along the thickness in Figure 11c,d. Multiple slips may cause the rearrangement and annihilation of dislocation due to the change of strain path, leading to the generation of cell blocks, as shown in Figure 12c. In addition, the {100} matrix became more active and many randomly arranged dense dislocation walls were formed, as shown in Figure 12d, because of activation of latent slip systems, which is beneficial to eliminate the orientation-dependent stored energy distribution.

### 4.2. Through-Thickness Stored-Energy Distribution 

As can be seen by combining Table 2 and Figure 4, the large stored energy gap between the {111} and {100} matrix was formed during the UR process because of the significant orientation-dependent difference in grain subdivision behavior. Many parallel MSBs existing in the {111} matrix, especially in the center region of the UR-Ta, can highly increase the stored energy and enlarge the orientation-dependent stored-energy difference. It can therefore be found that the energy stored in the center region was higher than at the surface, which is consistent with the hardness distribution of the deformed matrix seen in Figure 9a. On the contrary, grains are deformed more evenly, and a minor stored energy gap through the thickness is generated in the CR-Ta. The difference in stored energy between the {111} and {100} matrices was also narrowed conspicuously. The following reasons should account for the above difference between UR and CR sheets. Firstly, the starting material in this work was not uniform but revealed strong {100} and {111} texture in the surface and center regions, respectively [15]. The rolled tantalum single crystals experiment [38] indicates that the {111} orientation was more unstable when compared with the {100} orientation, which easily caused stress concentration and resulted in rotation and subdivision of the {111} matrix during the unidirectional rolling. Meanwhile, the fragmentation of the {111} matrix also promoted the split of its neighboring grain, such as the {100} matrix, due to the interaction of grain, leading to a relatively heavy deformation of grain in the center region. However, the coexisting reverse and orthogonal strain introduced by clock rolling may have enhanced the probabilities of movement, rearrangement, and annihilation of dislocation, contributing to the reduced dislocation plies-ups and the amount of MSBs, and thus decreasing the stored energy in the {111} matrix [19,39]. The {100} matrix in CR-Ta may be in a metastable state since more latent slip systems can be activated owing to the variation of rolling direction, leading to accumulation of dislocations and therefore increasing the stored energy of the {100} matrix. In addition, the direction of the shearing force varies inevitably with the change of strain path from passes to passes in the CR sheets, causing more homogeneous shearing deformation, while grains suffer more serious subdivision because of accumulative shearing force in UR sheets.

### 4.3. Through-Thickness Annealing Behavior of Ta Sheets with Different Rolling Methods

Obviously, there is a great difference in dislocation density and deformation substructure in the interior of grains with different orientation since the SFDR values vary with the local crystallographic orientation. Meanwhile, grains of the UR and CR sheets along the thickness also possess entirely different deformation behavior (Figure 10), which can be judged from the variance of SFDR values with the thickness. The kernel average misorientation (KAM) and grain reference orientation deviation-hyper (GROD-Hyper) further confirm these differences. Recrystallization usually consists of nucleation and subsequent grain growth, which is driven by the stored energy within the matrix. As shown in Table 2, there is a big stored energy gradient, though the thickness in the UR sheets and the energy stored in the center region are much higher than those in the surface region. The stored energy gap between the surface and center region is greatly narrowed by clock rolling, causing more homogenous distribution of stored energy along the thickness. In addition, the internal structure of the deformed matrix, as a nucleation site, also has a significant impact on determining the recrystallization behavior. MSBs and MBs in the {111} matrix of the UR-Ta are preferential places for nucleation upon annealing [40], and the stored energy as a driving force for the subsequent growth of nuclei is also much higher. Therefore, the defect-free grains with the {111} orientation first appear within the {111} matrix in the center region of UR sheets, which vanishes completely when annealed for 5 min, as shown in Figure 7. The {100} matrix gives rise to the low-fragmented regions and possesses low stored energy during deformation. Thus, unfavorable nucleation sites and the weak driving forces lead to sluggish recrystallization behavior in the {100} matrix. As the annealing time increases, it can be consumed by the subsequent growth of recrystallized grain consisting of the {111} orientation, primarily, leading to the occurrence of strong {111} texture. It is worth noting that the center region of the UR-Ta shows much easier and faster recrystallization than at the surface. However, the CR-Ta shows relatively homogeneous recrystallized dynamics along the thickness. The nucleation site and stored energy distribute more uniformly and recrystallized nuclei appear mainly along the {111} and {100} matricies or the {111}–{100} boundaries, as shown in Figure 8. In other words, the {100} matrix in the CR-Ta is more likely to provide nuclei sites as compared to that in the UR-Ta. Therefore, more homogeneous nuclei combined with a low stored energy are the driving forces contributing to the appearance of finer grains in fully recrystallized CR-Ta along the thickness.

## 5. Conclusions

The effects of clock rolling, which rotates the sample by 135° with respect to the previous rolling direction, on the deformed and recrystallized microstructure of tantalum sheets along the thickness were systematically investigated in the present study. The principle results can be drawn as follows:

1. More homogeneous γ and θ fibers along the thickness direction can be formed by clock rolling.
2. Many microshear bands and well-defined microbands occurred in the UR sheets, especially in the center region, while fragmentation within the {111} and {100} matrices in the CR sheets was more uniform along the thickness.
3. Both TEM and SFDR analysis show that there is more probability of activing the primary slip system of the matrix with the {111} orientation than with the {100} system, particularly in the center region, during the UR process. Deformation was relatively even, and multiple slip systems tended to be activated for the CR-Ta.
4. The stored energy gap between the {111} and {100} matrices is greatly narrowed by the CR process, and the stored energy distributes more uniformly along the thickness.
5. For the UR-Ta, the recrystallization was easier and faster in the center region than that of the surface region due to high stored energy, as the driving force, and preferential nucleation sites. The more homogeneous nucleating combined with a slower rate of grain growth resulted in finer grain size and smaller microstructure gradient for the annealed CR-Ta along the thickness.

## Figures and Tables

**Table 1:** The chemical composition of the original tantalum ingot (in wt. ppm).

C	N	H	O	Nb	Mo	W	Ti	Si	Fe	Ni	Ta
9	20	2	30	6.4	0.14	0.61	<0.001	<0.005	<0.005	<0.005	Balance

**Table 2:** The stored energy and relevant parameters used for the stored energy calculations.

	Position	Diffraction Plane/hkl	*Y*_hkl_	*ν*_hkl_	*B_r_*	*B_a_*	*SE_hkl_*	*SE*_222_/*SE*_200_
UR	Surface	{200}	145.517	0.316	0.179	0.100	3.63	2.36
		{222}	387.931	0.362	0.398	0.140	8.57	
	Center	{200}	145.517	0.316	0.192	0.100	4.43	2.87
		{222}	387.931	0.362	0.475	0.140	12.73	
CR	Surface	{200}	145.517	0.316	0.198	0.100	4.81	1.35
		{222}	387.931	0.362	0.354	0.140	6.53	
	Center	{200}	145.517	0.316	0.202	0.100	5.08	1.34
		{222}	387.931	0.362	0.361	0.140	6.84	

**Table 3:** Schmid factor difference ratio (SFDR) of some points as well as relative parameters used for the SFDR calculation.

			Point	Euler (*φ1, φ, φ2*)	Maximum (*S_M_*)	Secondary (*Ss*)	(*S_M_* − *S_S_*) × *S_M_*^−1^ (%)
UR	S	{100}	P1	279.43 43.889 70.950	0.2708	0.2609	3.64
			P2	277.96 43.529 71.340	0.272	0.2452	3.71
			P3	277.97 43.408 71.219	0.2731	0.2650	2.95
		{111}	P1	217.00 41.124 82.023	0.4623	0.3794	17.93
			P2	15.579 44.569 7.0144	0.4563	0.4083	10.5
			P3	15.650 44.848 7.6743	0.4567	0.4033	11.69
	C	{100}	P1	76.152 7.2152 17.893	0.2642	0.2521	4.55
			P2	116.82 16.695 64.727	0.2714	0.2591	4.53
			P3	115.14 17.019 68.883	0.2729	0.2564	6.03
		{111}	P1	26.542 45.999 15.271	0.4404	0.3548	19.43
			P2	24.906 43.064 17.406	0.4524	0.3688	18.47
			P3	25.559 37.626 12.854	0.4709	0.3867	17.87
CR	S	{100}	P1	269.98 4.0447 88.326	0.4109	0.3735	9.08
			P2	218.23 2.3379 45.701	0.4323	0.3907	9.60
			P3	226.42 2.8161 37.808	0.431	0.3903	9.43
		{111}	P1	160.81 36.475 62.089	0.4526	0.4181	7.62
			P2	158.93 35.937 61.261	0.4448	0.4193	5.73
			P3	157.42 36.953 62.397	0.4437	0.4148	6.51
	C	{100}	P1	144.99 25.852 28.954	0.4126	0.4031	2.29
			P2	139.21 23.775 31.780	0.4116	0.3980	3.28
			P3	137.89 23.296 33.902	0.4068	0.3934	3.27
		{111}	P1	354.46 35.820 56.002	0.4554	0.4228	10.47
			P2	354.42 35.617 54.992	0.4514	0.4085	9.50
			P3	353.94 35.341 55.239	0.4522	0.4104	9.24
